# Young-Onset Breast Cancer Outcomes by Time Since Recent Childbirth in Utah

**DOI:** 10.1001/jamanetworkopen.2022.36763

**Published:** 2022-10-14

**Authors:** Zhenzhen Zhang, Solange Bassale, Sonali Jindal, Alison Fraser, Emily Guinto, Weston Anderson, Motomi Mori, Ken R. Smith, Pepper Schedin

**Affiliations:** 1 Division of Oncological Sciences, Oregon Health & Science University, Portland; 2 Knight Cancer Institute, Oregon Health & Science University, Portland; 3 Biostatistics Shared Resources, Knight Cancer Institute, Oregon Health & Science University, Portland; 4 Department of Cell, Developmental & Cancer Biology, Oregon Health & Science University, Portland; 5 Pedigree and Population Resource, Population Sciences, Huntsman Cancer Institute, Salt Lake City, Utah; 6 Department of Biostatistics, St Jude Children’s Research Hospital, Memphis, Tennessee

## Abstract

**Importance:**

Breast cancer diagnosed within 5 to 10 years after childbirth, called *postpartum breast cancer* (PPBC), is associated with increased risk for metastasis and death. Whether a postpartum diagnosis is an independent risk factor or a surrogate marker of cancer features associated with poor outcomes remains understudied.

**Objective:**

To determine whether diagnostic temporal proximity to childbirth is associated with features of breast cancer associated with poor outcomes, including tumor stage, estrogen receptor (ER) status, and risk for distant metastasis and breast cancer–specific mortality, using a population database from the state of Utah.

**Design, Setting, and Participants:**

This population-based cohort study using the Utah Population Database (UPDB) included individuals with stage I to III breast cancer diagnosed at age 45 years or younger between 1996 and 2017, followed-up until February 2020. Participant data were analyzed from November 2019 to August 2022.

**Exposure:**

The primary exposures were no prior childbirth or time between most recent childbirth and breast cancer diagnosis. Patients were grouped by diagnoses within less than 5 years, 5 to less than 10 years, or 10 years or more since recent childbirth.

**Main Outcomes and Measures:**

The 2 primary outcomes were distant metastasis–free survival and breast cancer–specific death. Cox proportional hazard models were used to investigate associations between exposures and outcomes adjusting for diagnosis year, patient age, tumor stage, and estrogen receptor (ER) status.

**Results:**

Of 2970 individuals with breast cancer diagnosed at age 45 years or younger (mean [SD] age, 39.3 [5.0] years; 12 Black individuals [0.4%], 2679 White individuals [90.2%]), breast cancer diagnosis within 5 years of recent childbirth was independently associated with approximately 1.5-fold elevated risk for metastasis (hazard ratio [HR], 1.5; 95% CI, 1.2-2.0) and breast cancer–specific death (HR, 1.5; 95% CI, 1.1-2.1) compared with nulliparous individuals. For cancers classically considered to have tumor features associated with good outcomes (ie, stage I or II and ER-positive), a postpartum diagnosis was a dominant feature associated with increased risk for metastasis and death (eg, for individuals with ER-positive disease diagnosed within <5 years of childbirth: age-adjusted metastasis HR, 1.5; 95% CI, 1.1-2.1; *P* = .01; age-adjusted death HR, 1.5; 95% CI, 1.0-2.1; *P* = .04) compared with nulliparous individuals. Furthermore, liver metastases were specifically increased in the group with diagnosis within 5 years postpartum and with positive ER expression (38 of 83 patients [45.8%]) compared with the nulliparous (28 of 77 patients [36.4%]), although the difference was not statistically significant. Overall, these data implicate parity-associated breast and liver biology in the observed poor outcomes of PPBC.

**Conclusions and Relevance:**

In this cohort study of individuals with breast cancer diagnosed at age 45 years or younger, a postpartum breast cancer diagnosis was a risk factor associated with poor outcomes. Irrespective of ER status, clinical consideration of time between most recent childbirth and breast cancer diagnosis could increase accuracy of prognosis in patients with young-onset breast cancer.

## Key Points

**Question:**

Is a postpartum breast cancer diagnosis independently associated with risk for breast cancer metastasis or breast cancer–specific mortality?

**Findings:**

In this cohort study including 2970 patients diagnosed with breast cancer at age 45 years or younger, a breast cancer diagnosis within less than 5 years post partum was associated with elevated risk for metastasis and breast cancer–specific death compared with nulliparous patients with breast cancer, independent of estrogen receptor status or tumor stage. There was increased lymph node involvement and liver metastasis in patients with estrogen receptor–positive disease diagnosed less than 5 years postpartum.

**Meaning:**

These findings suggest that a postpartum breast cancer diagnosis was an independent risk factor associated with breast cancer progression and implicates postpartum biology in the increased metastasis, including postpartum liver biology.

## Teaser

This cohort study assesses breast cancer outcomes by time from childbirth to breast cancer diagnosis in patients aged 45 years or younger.

## Introduction

Malignant young-onset breast cancer (YOBC), diagnosed at age 15 to 44 years, accounted for approximately 12% of all new breast cancer diagnoses in the United States from 1975 to 2018,^1^ with significantly higher incidences reported in other countries,^2,3^ including European countries.^4^ Furthermore, incidence of YOBC is increasing worldwide.^5,6,7,8^ A recent retrospective Surveillance, Epidemiology, and End Results (SEER) registry study representing approximately 28% of the US population reported annual increases in the incidence of YOBC between 2000 and 2015, with a 1.62% annual increase in people aged 20 to 29 years, a 0.31% increase in people aged 30 to 39 years, and 0.34% increase in people aged 40 to 49 years.^8^ Another study among individuals with breast cancer in Connecticut showed the incidence of breast cancer for individuals aged 25 to 39 years increased 0.65% each year from 1935 to 2015.^6^ This increasing incidence does not appear to be due to increased breast cancer screening, as most cases were among individuals too young for routine screening.^9,10^ Additionally, the reduction in mean family size over time has not been found to explain increased incidence in younger individuals.^6^ Importantly, compared with later-age–onset breast cancers, YOBC is enriched in poor prognostic tumor features, including increases in estrogen receptor (ER)–negative tumors,^11,12,13,14^ has higher levels of mortality,^14,15,16,17^ and has experienced limited gains in treatment efficacy.^17^

In young individuals, breast cancer is often diagnosed in proximity to a recent childbirth, and the role of childbirth or proximity to childbirth in mediating increased risk and poor outcomes in breast cancer among young people are active areas of research. Robust population-level data show that childbirth has a dual association with risk. Early age at first childbirth is associated with a transient increased risk of developing breast cancer of either ER status in the first decade after childbirth. This transient increased risk is associated with protection in later years for ER-positive breast cancer; however, no crossover to a protective association was observed in ER-negative breast cancer compared with nulliparous individuals.^18,19^ These results are similar to those reported in a 2022 meta-analysis^20^ that showed 0 to 10 years postpartum as a high-risk window for YOBC, especially after a second childbirth. With respect to breast cancer outcomes, the relatively rare breast cancers diagnosed and treated during pregnancy, which account for approximately 4% of YOBC, are reported to have outcomes similar to those in nulliparous individuals.^21^ Conversely, meta-analyses of YOBC find a postpartum diagnosis (within 5-10 years of childbirth) to be consistently associated with increased risk of distant metastasis and death.^22,23,24^ Given that more than 50% of all YOBC is diagnosed within 10 years of childbirth,^25^ the concept that postpartum biology plays a significant role in metastasis in YOBC requires further investigation. The disparate impacts of a diagnosis during pregnancy compared with recently postpartum has led to recent recommendations for the separate study of breast cancer during pregnancy and postpartum breast cancer (PPBC).^26^ In this study with a focus on PPBC, we sought to better understand whether temporal proximity to childbirth was associated with key prognostic features of breast cancer, including tumor stage and estrogen receptor (ER) status, as well as risk of distant metastasis and breast cancer–specific mortality. If proximity to a recent childbirth is an independent risk factor, then current clinical guidelines reliant on tumor staging and biologic subtyping alone may be insufficient to accurately project the risk of recurrence and optimal treatment strategies in young patients.

## Methods

This cohort study was deemed exempt from review and informed consent by The University of Utah and the Oregon Health & Science University institutional review boards because it was deemed nonhuman research. This study is reported following the Strengthening the Reporting of Observational Studies in Epidemiology (STROBE) reporting guideline.

### Database Setting

The study population was identified from the Utah Population Database (UPDB), which integrates patient-level data between Utah Vital Records, including birth and death; Utah Cancer Registry, a SEER Cancer Registry; the University of Utah Health Sciences Center (UUHSC); and Intermountain Healthcare (IMHC). Combined, UUHSC and IMHC provide care to greater than 85% of the Utah population. The UPDB is updated annually, and probabilistic record linking is performed for each individual case in the database. Because of its size and the varied sources of information, most families living in Utah are represented.

### Participants

We identified all 56 153 non–distant-metastatic breast cancer cases in the UPDB, defined as SEER summary stage 0 to 4 breast cancer. A total of 8526 patients diagnosed at ages older than 15 years and up to 45 years were included. Because the Utah Ambulatory Surgery and Utah Inpatient Hospital Discharge data were available in UPDB beginning in 1996, 4376 patients diagnosed since 1996 were included. Patients were subsequently excluded due to unknown and/or late stage and due to being missing from the Utah Cancer Registry (UCR), leaving 4062 patients aged up to 45 years diagnosed with localized or regional breast cancers between January 1, 1996, and December 31, 2017. Next, we excluded 299 patients with 2 or more primary cancers, 207 patients with 2 or more breast cancers diagnosed more than 3 months apart, 579 patients with in situ breast cancers, and 7 patients with primigravid breast cancer (ie, breast cancer diagnosed during first pregnancy). The final analysis set included 2970 patients, including 860 nulliparous patients (ie, those who never had childbirth) and 2110 parous patients (eFigure 1 in the Supplement). Parous patients were further categorized based on time from most recent childbirth to breast cancer diagnosis into the following PPBC categories: diagnosed within less than 5 years (614 individuals); 5 to less than 10 years (615 individuals); or 10 years or longer (881 individuals) from recent childbirth. Patients were followed up to February 2020 (cutoff date) or until death. Race and ethnicity data were based on abstractions from clinical records and multiple sources from the Utah Population Database (eg, driver’s license, voting records, vital records) and were then standardized and categorized into the National Institutes of Health categories, including Black, White, or others. Patients in the other race and ethnicity category included American Indian, Asian, Native Hawaiian, and multiple races. Race and ethnicity were assessed because socioeconomic status is a major source for breast cancer inequality, as it affects both effective diagnosis and treatment. Individuals with low socioeconomic status are more likely to be Black or Hispanic and may lack consistent access to care. This factor can delay testing and medical care. Collectively, this results in fewer breast cancer examinations and a higher chance of a metastatic breast cancer diagnosis. At the same time, Black and Hispanic individuals have higher fertility rates. Overall, race and ethnicity is a potential confounder for the association between fertility schedules and metastatic breast cancer. For example, the 2014 POSH study^27^ found that Black individuals were more likely to have distant metastasis at presentation compared with White individuals (5.1% vs 2.4%).

### Outcomes, Exposures, and Covariates

The primary outcomes for this study are distant metastasis and breast cancer–specific death. Distant metastases were defined as metastasis to organs outside of the ipsilateral breast and local draining lymph nodes following a primary diagnosis of nonmetastatic breast cancer. Covariates included tumor size, stage, lymph node involvement, and ER status. Distant metastasis survival was defined as the time from the initial diagnosis to the first documented distant metastasis or death, whichever occurred first. Those without distant metastasis or death were censored at the date of last contact, up to the study cutoff date (February 2020). Breast cancer–specific survival was defined as the time from the initial diagnosis to the date of death due to breast cancer. Those without death due to breast cancer were censored at the date of last contact or the date of death due to other causes, up to the study cutoff date (February 2020). The underlying cause of death for deceased patients was obtained via the annual linkages with Utah state death certificates and stored in the Utah SEER database. The event for breast cancer–specific death was based on the SEER cause-specific death classification using the *International Statistical Classification of Diseases and Related Health Problems, Tenth Revision *(*ICD-10*).

The main postpartum interval groups used in the comparison are nulliparous and PPBC less than 5 years, PPBC 5 to less than 10 years, and PPBC 10 years or longer, previously identified postpartum time frames associated with higher risk for YOBC.^22,23,24,25,28^ Clinical prognostic factors include patient age at diagnosis, tumor ER status, lymph node involvement, tumor size, American Joint Committee on Cancer (AJCC) stage, biologic subtype (defined by ER, progesterone receptor [PR], and human epidermal growth factor receptor 2 [ERBB2; formerly HER2]) status, year of diagnosis, sites of distinct metastasis, and first site of distant metastasis. AJCC stage was derived from SEER Summary staging using the Adjusted AJCC 6th Stage algorithm.^29^

### Statistical Analysis

Time since recent childbirth groups were compared by performing Pearson χ^2^ tests for each categorical variable and the Kruskal-Wallis test for the continuous variables to identify significant differences among the 4 groups. The Kaplan-Meier method was used to calculate survival estimates, and the log-rank test was used to compare the survival curves. The Cox proportional hazards regression was applied to identify factors associated with the risk of distant metastasis–free survival and to estimate hazards for breast cancer–specific death. The proportionality assumption was tested using the Schoenfeld residuals, with ER status showing a nonconstant hazard over time. To account for this, the data were stratified by ER status where appropriate. The models are initially based on age-adjusted associations of the time interval since recent childbirth groups, followed by multivariate models that include covariates time interval since recent childbirth, diagnosis year, patient age at diagnosis, stage, and breast cancer ER status. Hazard ratios (HRs) and 95% CIs were calculated correspondingly.

A sensitivity analysis was conducted to compare distant metastasis–free survival and breast cancer–specific death among the 4 time intervals since recent childbirth groups stratified by stage (AJCC stage I, II, III). A second sensitivity analysis examined the interaction between ER status and time since recent childbirth group in association with distant metastasis–free survival and breast cancer–specific death.

Additionally, we conducted descriptive analysis of distant metastasis by organ site. Among patients with information on the first site of distant metastasis, we also compared the frequency distribution of organ site by time since recent childbirth groups. All the statistical analyses were conducted using SAS software version 9.4 (SAS Institute). Tests of statistical significance were determined using 2-tailed tests, and *P* < .05 was considered statistically significant. The first site of metastasis study was not powered to explore the ER-negative only breast cancer groups. Data were analyzed from November 2019 to August 2022.

## Results

### Patient Characteristics

Of 56 153 patients with nonmetastatic invasive breast cancer in the Utah Cancer Registry, 18% were aged 45 years or younger at diagnosis, higher than the national mean of 12%.^1^ After excluding ineligible patients, our final analytic cohort included 2970 patients diagnosed between 1996 and 2017 with stage I, II, or III breast cancer (mean [SD] age, 39.3 [5.0] years; 12 Black individuals [0.4%], 2679 White individuals [90.2%]). The clinical and demographic characteristics of the cohort, stratified by time since recent childbirth group, are summarized in Table 1. Across the time since recent childbirth groups, there were differences in age at diagnosis, race, ER status, tumor size, lymph node, stage, biologic subtype, year of diagnosis distribution, and number of patients with distant metastasis. For race, the nulliparous group had the lowest proportion of White individuals (748 of 860 individuals [87.0%]) compared with the other groups (Table 1). For tumor characteristics, the group with PPBC within less than 5 years had the greatest proportion of ER-negative tumors (160 of 597 individuals [26.8%]), larger tumor size (86 of 584 individuals [14.7%] with tumors >5 cm), more lymph node involvement (360 of 597 individuals [60.3%]), and higher stage (103 of 502 individuals [20.5%] with stage III) compared with other groups. The group with PPBC within less than 5 years also had fewer patients with Luminal A subtype (174 of 295 patients [59.0%]) and more patients whose tumors advanced to distant metastasis (118 of 614 individuals [19.2%]) compared with the other groups (Table 1).

**Table 1.** Demographic and Clinical Data of Analytic Cohort

Characteristic	No. (%) (N = 2970)^a^			
	Nulliparous	PPBC <5 y	PPBC 5 to <10 y	PPBC ≥10
No. (%)	860 (29.0)	614 (20.7)	615 (20.7)	881 (29.7)
Mean age at diagnosis (SD), y	38.9 (5.7)	35.6 (5.0)	39.5 (4.1)	42.2 (2.7)
Race^b^				
Black	10 (1.2)	0	1 (0.2)	1 (0.1)
White	748 (87.0)	554 (90.2)	558 (90.7)	819 (93.0)
Other	102 (11.2)	60 (9.8)	56 (9.1)	61 (6.9)
Estrogen receptor status				
Positive	637 (78.0)	437 (73.2)	467 (78.6)	662 (77.6)
Negative	180 (22.0)	160 (26.8)	127 (21.4)	191 (22.4)
Missing	43	17	21	28
Tumor size, cm				
0.1 to 2.0	443 (53.8)	259 (44.3)	320 (54.1)	494 (58.5)
>2.0 to 5.0	305 (37.0)	239 (40.9)	220 (37.2)	306 (36.2)
>5.0	76 (9.2)	86 (14.7)	52 (8.8)	45 (5.3)
Missing	36	30	23	36
Lymph node involvement				
Yes	378 (45.8)	360 (60.3)	257 (43.0)	351 (40.9)
No	448 (54.2)	237 (39.7)	341 (57.0)	507 (59.1)
No nodes examined	34	17	17	23
Stage				
I	330 (47.1)	158 (31.5)	246 (46.7)	391 (53.2)
II	293 (41.8)	241 (48)	207 (39.3)	270 (36.7)
III	78 (11.1)	103 (20.5)	74 (14.0)	74 (10.1)
Missing	159	112	88	146
Biologic subtype				
Luminal A^c^	211 (64.7)	174 (59.0)	196 (70.0)	264 (68.6)
Luminal B^d^	61 (18.7)	62 (21.0)	36 (12.9)	52 (13.5)
ERBB2+^e^	19 (5.8)	21 (7.1)	20 (7.1)	18 (4.7)
Triple negative^f^	35 (10.7)	38 (12.9)	28 (10.0)	51 (13.2)
Unknown ERBB2	520	314	331	493
Unknown or other	14	5	4	3
Year of diagnosis				
1996-1998	94 (10.9)	49 (8.0)	60 (9.8)	100 (11.4)
1999-2004	208 (24.2)	119 (19.4)	120 (19.5)	221 (25.1)
2005-2017	558 (64.9)	446 (72.6)	435 (70.7)	560 (63.6)
Patients with metastasis^g^	98 (11.4)	118 (19.2)	80 (13.0)	100 (11.4)

Abbreviations: +, positive; ER, estrogen receptor; ERBB2, human epidermal growth factor receptor 2 (formerly HER2/neu); PPBC, postpartum breast cancer (expressed as years from childbirth to breast cancer diagnosis).

^a^ All the group comparisons for variables listed here had *P* < .05. Missing categories were excluded from statistical analysis.

^b^ Patients were categorized as Black, White, or others based on the NIH Race Data. Others include Asian, American Indian, Native Hawaiian, and multiple races.

^c^ Defined as ER-positive, progesterone receptor positive or negative, and ERBB2-negative.

^d^ Defined as ER-positive, progesterone receptor positive or negative, and ERBB2-positive.

^e^ Defined as ER-negative and progesterone receptor negative.

^f^ Defined as ER-negative, progesterone receptor negative, and ERBB2-negative.

^g^ A total of 396 patients had distant metastasis, with 387 patients with known first site of distant metastasis.

### Association of Childbirth and Time Since Recent Childbirth With Breast Cancer Outcomes

**Figure 1.**
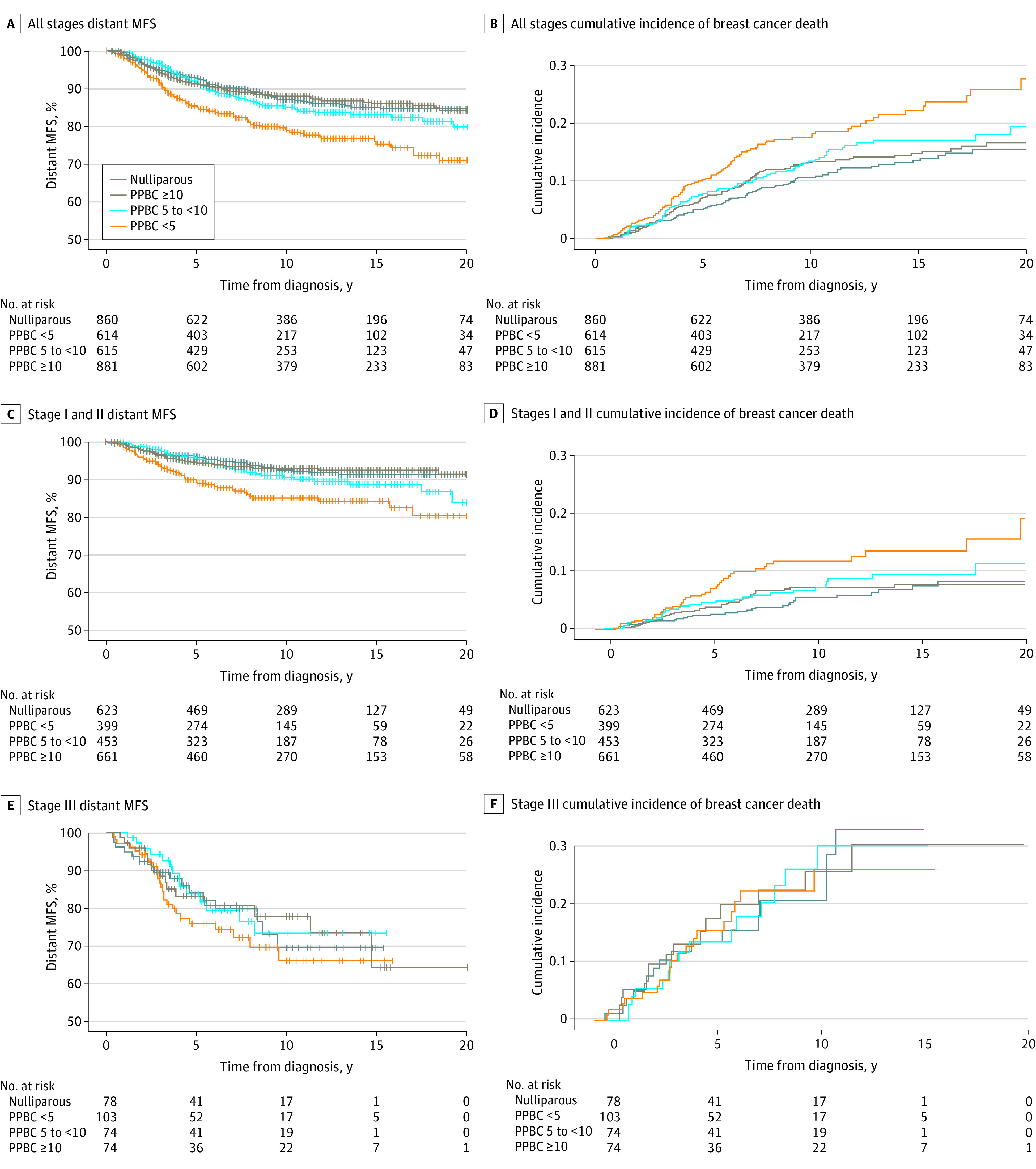
Long-term Outcomes by Stage and Time Since Recent Childbirth Group MFS indicates metastasis-free survival; PPBC, postpartum breast cancer expressed as time from childbirth to breast cancer diagnosis in years.

We evaluated the association of time since recent childbirth with risk of developing breast cancer distant metastasis. We examined the percentage of individuals developing distant metastases across the entire cohort, of whom 396 of 2970 individuals (13.3%) had cancers that advanced to distant metastases. We observed that a diagnosis of breast cancer within less than 5 years of childbirth was associated with an increased risk of breast cancer distant metastasis (age-adjusted HR, 1.7; 95% CI, 1.3-2.2; *P* < .001) compared with nulliparous individuals (Table 2; Figure 1A). This increased risk persisted after controlling for year of diagnosis (as a proxy for changes in treatment over time), patient age, tumor stage, and ER status (multivariate HR, 1.5; 95% CI, 1.2-2.0; *P* = .002), identifying proximity to childbirth as an independent risk factor (Table 2). Similarly, a diagnosis within less than 5 years of childbirth increased risk for breast cancer–specific death in age-adjusted (HR, 1.7; 95% CI, 1.3-2.3; *P* < .001) and multivariate (HR, 1.5; 95% CI, 1.1-2.1; *P* = .004) analyses (Table 2; Figure 1B).

**Table 2.** Cox Proportional Hazard Regression Models for the Associations of Time From Recent Childbirth With Distant Metastasis and Breast Cancer–Specific Mortality

PPBC time, y	Total, No. (PY)	Mortality rate, No./1000 PY	Distant metastasis						Breast cancer–specific mortality					
			Age-adjusted			Multivariate			Age-adjusted			Multivariate		
			HR (95% CI)	*P* value	Overall *P* value	HR (95% CI)	*P* value	Overall *P* value	HR (95% CI)	*P* value	Overall *P* value	HR (95% CI)	*P* value	Overall *P* value
All stages^a^														
Nulliparous	860 (5440)	10.3	1 [Reference]	NA	.002	1 [Reference]	NA	.02	1 [Reference]	NA	.007	1 [Reference]	NA	.04
<5	614 (5882)	18.8	1.7 (1.3-2.2)	<.001		1.5 (1.2-2.0)	.002		1.7 (1.3-2.3)	<.001		1.5 (1.1-2.1)	.004	
5 to <10	615 (8754)	13.1	1.2 (0.9-1.6)	.26		1.2 (0.9-1.6)	.25		1.3 (0.9-1.7)	.12		1.3 (0.9-1.7)	.11	
≥10	881 (8561)	11.5	1.1 (0.8-1.5)	.49		1.1 (0.8-1.5)	.57		1.2 (0.9-1.6)	.21		1.2 (0.9-1.6)	.32	
Stage I-II^b^														
Nulliparous	623 (6144)	5.0	1 [Reference]	NA	.01	1 [Reference]	NA	.03	1 [Reference]	NA	.02	1 [Reference]	NA	.04
<5	399 (3501)	11.7	2.0 (1.3-3.0)	.002		1.8 (1.2-2.8)	.004		2.1 (1.3-3.4)	.002		2.0 (1.2-3.2)	.005	
5 to <10	453 (4237)	7.3	1.4 (0.9-2.2)	.17		1.4 (0.9-2.2)	.15		1.5 (0.9-2.4)	.14		1.5 (0.9-2.4)	.13	
≥10	661 (6398)	5.9	1.2 (0.8-1.9)	.46		1.2 (0.8-1.9)	.45		1.3 (0.8-2.1)	.32		1.2 (0.7-2.	.48	
Stage III^b^														
Nulliparous	78 (535)	29.9	1 [Reference]	NA	.78	1 [Reference]	NA	.77	1 [Reference]	NA	.96	1 [Reference]	NA	.99
<5	103 (695)	27.3	1.2 (0.7-2.3)	.50		1.3 (0.7-2.3)	.48		0.9 (0.4-1.7)	.71		0.9 (0.5-1.7	.73	
5 to <10	74 (533)	28.1	0.9 (0.4-1.8)	.76		0.9 (0.4-1.8)	.76		0.9 (0.5-1.9)	.88		0.9 (0.4-1.8	.79	
≥10	74 (532)	28.2	1.0 (0.5-2.1)	.98		1.0 (0.5-2.1)	.99		1.1 (0.5-2.2)	.86		0.9 (0.4-2)	.87	
ER+^c^														
Nulliparous	637 (6180)	10.4	1 [Reference]	NA	.02	1 [Reference]	NA	.14	1 [Reference]	NA	.18	1 [Reference]	NA	.40
<5	437 (3770)	17.0	1.5 (1.1-2.1)	.01		1.4 (1.0-1.9)	.06		1.5 (1.0-2.1)	.04		1.3 (0.9-1.9)	.15	
5 to <10	467 (4370)	11.9	1.1 (0.8-1.5)	.63		1.1 (0.8-1.5)	.58		1.2 (0.8-1.7)	.41		1.2 (0.8-1.7)	.38	
≥10	662 (6552)	9.2	0.9 (0.6-1.3)	.58		0.9 (0.6-1.3)	.54		1.0 (0.7-1.5)	.96		1.0 (0.7-1.4)	.89	
ER−^c^														
Nulliparous	180 (1756)	11.4	1 [Reference]	NA	.03	1 [Reference]	NA	.04	1 [Reference]	NA	.03	1 [Reference]	NA	.04
<5	160 (1418)	25.4	2.5 (1.3-4.7)	.005		2.4 (1.3-4.7)	.007		2.4 (1.3-4.2)	.003		2.3 (1.3-4.1)	.004	
5 to <10	127 (1242)	16.9	1.4 (0.7-2.8)	.40		1.4 (0.7-2.9)	.35		1.4 (0.8-2.6)	.27		1.5 (0.8-2.8)	.20	
≥10	191 (1835)	19.1	1.8 (0.9-3.4)	.09		1.8 (1.0-3.5)	.06		1.5 (0.8-2.6)	.17		1.6 (0.9-2.8)	.11	

Abbreviations: −, negative; +, positive; ER, estrogen receptor; HR, hazard regression; PPBC, postpartum breast cancer (expressed as years from childbirth to breast cancer diagnosis); NA, not applicable; PY, person-years.

^a^ Multivariate models for all cases were adjusted for diagnosis year (1996-1998, 1999-2004, or 2005-2017), diagnosis age (continuous), American Joint Committee on Cancer Stage (I, II, III, or unknown), ER status (ER+, ER−, or unknown) (eTable 3 in the Supplement).

^b^ Multivariate models for specific stages were adjusted for diagnosis year (1996-1998, 1999-2004, or 2005-2017), diagnosis age (continuous), and ER status (ER+, ER−, and unknown).

^c^ Multivariate models for ER status were adjusted for diagnosis year (1996-1998, 1999-2004, or 2005-2017), diagnosis age (continuous), and American Joint Committee on Cancer Stage (I, II, III, or unknown).

Next, we evaluated outcomes by stage at diagnosis. We found that individuals diagnosed within less than 5 years of childbirth with stage I and II disease had more than 2-fold increased risk of distant metastasis (age-adjusted HR, 2.0; 95% CI, 1.3-3.0; *P* = .002) and breast cancer–specific death (age-adjusted HR, 2.1; 95% CI, 1.3-3.4; *P* = .002) compared with nulliparous individuals (Table 2; Figure 1C and D). These risks persisted after controlling for year of diagnosis, patient age, tumor stage, and ER status (metastasis: multivariate HR, 1.8; 95% CI, 1.2-2.8; *P* = .004; breast cancer–specific death: multivariate HR, 2.0; 95% CI, 1.2-3.2; *P* = .005) (Table 2). In contrast, we did not observe an increased risk of distant metastasis or breast cancer–specific death in patients with stage III disease diagnosed within less than 5 years of childbirth; however, stage III diagnoses were less frequent (329 patients [13.4%]) in our cohort, and the analyses may be underpowered (Table 2; Figure 1E and F).

### ER-Negative Tumors in PPBC

We next evaluated whether the increases in distant metastasis and breast cancer–specific death observed in the group with PPBC within less than 5 years were associated with enrichment of poor prognostic tumor subtypes, such as ER-negative disease, which is elevated in YOBC compared with older-onset breast cancer.^11,15^ Notably, in this Utah cohort, most patients diagnosed in the less than 5 years group had ER-positive disease (437 of 597 individuals [73.2%]). We found that the group with PPBC within less than 5 years had more ER-negative disease (160 of 597 individuals [26.8%]) compared with the other 3 groups (nulliparous: 180 of 817 individuals [22.0%]; 5 to <10 years: 127 of 594 individuals [21.4%]; ≥10 years: 191 of 853 individuals [22.4%]) (Table 1), with the difference mainly driven by the lower median age of the group with PPBC within less than 5 years (eTable 1 in the Supplement). When we stratified the data by age (≤36 vs >36 years) for each of the PPBC groups, we found that the proportion of ER-negative disease was increased in younger individuals in every PPBC category (eTable 1 in the Supplement). In other words, in each reproductive category, ER-negative disease was more prevalent in the younger age group. At the same time, the overall proportion of ER-negative disease in the group aged 36 years or younger was fairly consistent across all reproductive categories (Table 1). However, in the group aged 36 years or younger, the proportion of ER-negative disease was increased in the group with PPBC within less than 5 years compared with the other groups (eTable 1 in the Supplement). In support of ER status not being the primary driver of distant metastasis in this cohort of younger individuals, the overall proportion of patients progressing to distant metastasis was the same regardless of ER status, with 282 of 2203 patients (12.8%) with ER-positive disease and 88 of 658 patients (13.4%) with ER-negative disease progressing to distant metastasis (*P* = .70) (eTable 2 in the Supplement). Furthermore, when both ER status and proximity to recent childbirth were included in the same multivariate model, we found that ER status was not associated with distant metastatic disease, whereas temporal proximity to childbirth was associated (Table 2 and eTable 3 in the Supplement). Of note, since nearly 80% of all cancers were ER-positive, 3 times more individuals with ER-positive disease advanced to distant metastasis than individuals with ER-negative disease (eTable 2 in the Supplement). Rigorous evaluation of ERBB2 status by reproductive category was not possible due to the lack of ERBB2 clinical data for most patients (Table 1).

### Association of Time Since Recent Childbirth With Breast Cancer Outcomes by ER Status

**Figure 2.**
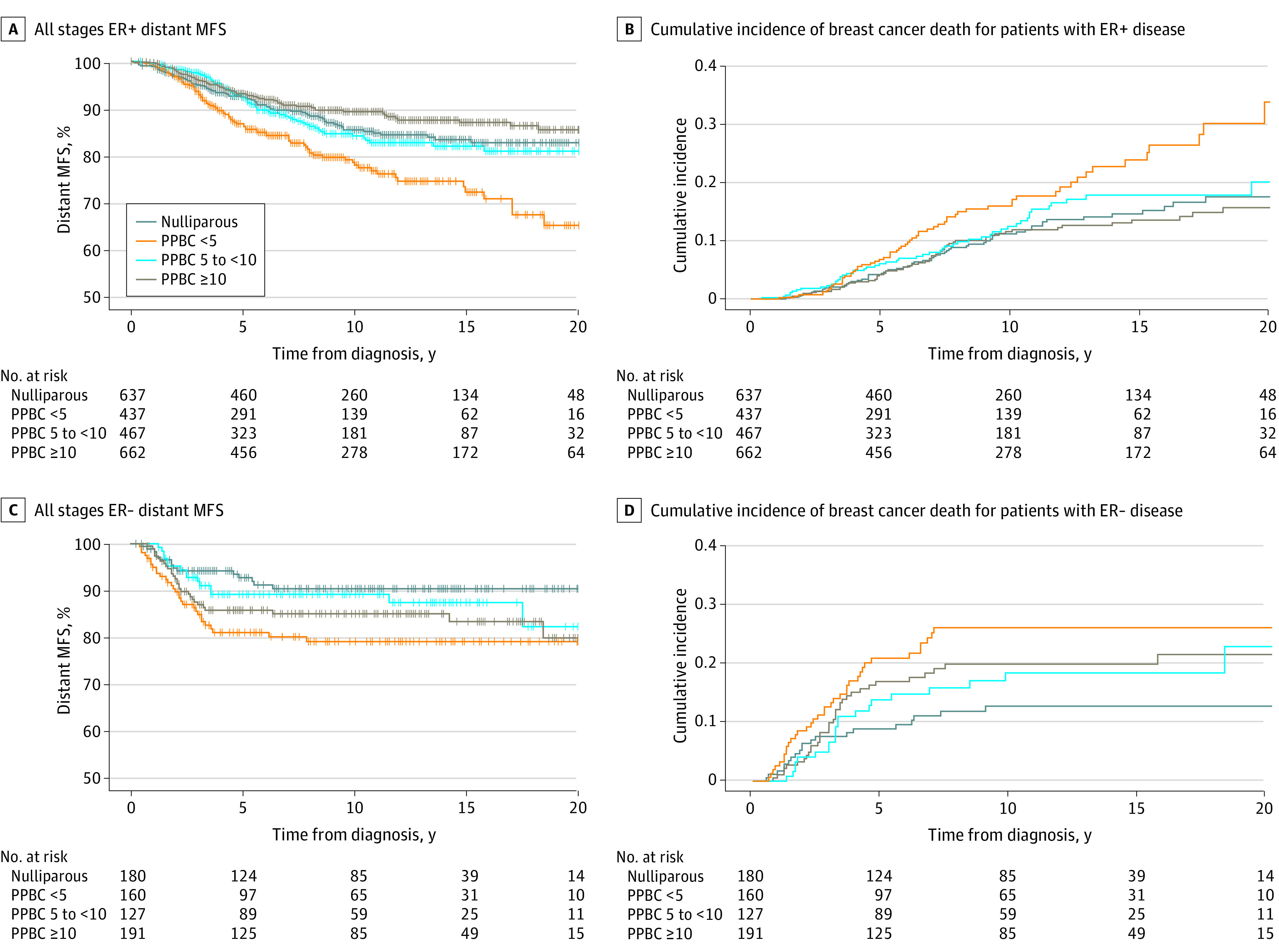
Long-term Outcomes by Estrogen Receptor (ER) Status and Time Since Recent Childbirth Group This analysis only included 2891 patients with known ER status. − indicates negative; +, positive; MFS, metastasis-free survival; PPBC, postpartum breast cancer expressed as time from childbirth to breast cancer diagnosis in years.

We next evaluated the association of a recent childbirth with disease outcomes separately for ER-positive and ER-negative disease. Among individuals with ER-positive disease, compared with nulliparous individuals, we found individuals diagnosed within less than 5 years of childbirth were 1.5 times as likely to progress to distant metastasis (age-adjusted HR, 1.5; 95% CI, 1.1-2.1; *P* = .01) and die from their disease (age-adjusted HR, 1.5; 95% CI, 1.0-2.1; *P* = .04) (Table 2; Figure 2A and B). Following adjustment for year of diagnosis, patient age, and tumor stage, the risk for distant metastasis and breast cancer–specific death among individuals with ER-positive disease diagnoses within less than 5 years from childbirth no longer met statistical significance. This loss of significance was primarily driven by stage migration (Table 1 and Table 2). Further investigation into this stage migration revealed that increases in tumor size for the group with PPBC within less than 5 years were modest (eFigure 2 in the Supplement), and that increased lymph node involvement was the primary reason for increased stage at time of diagnosis. Lymph node involvement was observed in 360 of 597 individuals (60.3%) in the PPBC within less than 5 years group, compared with 378 of 826 individuals (45.8%) in the nulliparous group, 257 of 598 individuals (43.0%) in the 5 to less than 10 years group, and 351 of 858 individuals (40.9%) in the 10 years or longer group (Table 1), corroborating and expanding on previous reports of increased lymphangiogenesis in PPBC.^30,31^

Patients with ER-negative disease diagnosed in the less than 5 years period were approximately 2.5 times as likely to progress to distant metastatic disease (age-adjusted HR, 2.5; 95% CI, 1.3-4.7; *P* = .005) and die from breast cancer (age-adjusted HR, 2.4; 95% CI, 1.3-4.2; *P* = .003) compared with nulliparous patients with ER-negative disease (Table 2; Figure 2C and D). For patients with ER-negative disease, this increased risk for distant metastasis and breast cancer–specific death persisted after controlling for year of diagnosis, age at diagnosis, and tumor stage (metastasis: multivariate HR, 2.4; 95% CI, 1.3-4.7; *P* = .007; breast cancer–specific death: multivariate HR, 2.3; 95% CI, 1.3-4.1; *P* = .004) (Table 2).

### Patterns of Distant Metastasis in YOBC

**Figure 3.**
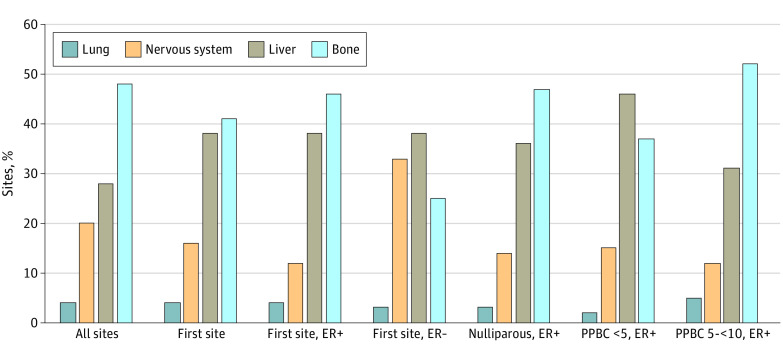
Sites of Metastasis by Estrogen Receptor (ER) Status and Time Since Recent Childbirth Group Analyses included a total of 1787 known metastasis sites (including multiple metastasis sites per person of 396 patients), 387 patients with known first site of metastasis, 282 patients with ER-positive (ER+) disease, 88 patients with ER-negative (ER−) disease, 77 nulliparous patients with ER+ disease, 83 patients with ER+ disease diagnosed within less than 5 years of childbirth (PPBC <5), and 58 patients with ER+ disease diagnosed at 5 to less than 10 years since childbirth (PPBC 5-<10).

We next evaluated all sites of metastasis. In our cohort of 2970 patients, 396 were reported to advance to metastasis, with subsequent metastasis sites followed over time until censoring date (death, loss to follow-up, or February 2020). A total of 1787 metastatic lesions were detected across the study period. When examining these 1787 distant metastatic sites in these 396 patients, we found that bone was the most frequent distant metastatic site (860 sites [48.1%]), followed by liver (504 sites [28.2%]), nervous system (355 sites [19.9%]), lung (63 sites [3.5%]) and kidney (5 sites [<0.1%]) (Figure 3A).

We next assessed 387 patients with known first site of distant metastasis. We found the share of first site of metastasis for brain, lung and kidney remained essentially unchanged between all sites and first sites of distant metastasis (Figure 3B). However, we found the share of bone as first site of metastasis decreased from 48.1% to 41.3% (160 sites) and liver increased from 28.2% to 37.7% (146 sites) (Figure 3B). Combined, these data suggest bone, liver, and nervous system were preferred first and all sites of distant metastasis in this large cohort of YOBC.

We next delineated first site of distant metastasis by ER status and found differential patterns of metastatic spread (eTable 2 in the Supplement). Of 387 patients with known first site of distant metastasis, 370 had known ER status. Most of these patients (282 patients [76.2%]) had ER-positive disease, and 88 patients (23.8%) had ER-negative disease. The subset analysis of first site of distant metastases for ER-positive disease was similar to the overall cohort, which may be expected, given that more than 70% of patients with distant metastases had ER-positive disease (Figure 3C). However, we did observe an increase in first site of distant metastasis to the bone and a decrease in brain metastasis in patients with ER-positive disease (Figure 3C). Conversely, the pattern of first site of distant metastasis in ER-negative disease differed from the overall cohort pattern. Specifically, the ER-negative group was enriched for nervous system metastases, and had substantially fewer bone metastases (Figure 3D). Overall, these data are consistent with previous reports of ER status in association with site of distant metastasis.^32,33^

### Pattern of First Site of Distant Metastasis by Time Since Recent Childbirth Status

The ER-positive YOBC cohort was sufficiently large to examine whether time since recent childbirth status was associated with first site of metastasis. Among patients with ER-positive disease, we found that the PPBC within less than 5 years group had a distinct metastasis pattern, although there was no statistically significant difference. Specifically, we found the PPBC within less than 5 years group had a greater proportion of liver metastasis (38 of 83 patients [45.8%]) compared with the nulliparous group (28 of 77 patients [36.4%]) and PPBC at 5 to less than 10 years group (18 of 58 patients [31.0%]) (*P* = .08). The PPBC within less than 5 years group also had a smaller proportion of bone metastasis (31 of 83 patients [37.3%]) compared with the nulliparous group (36 of 77 patients [46.8%]) and the PPBC at 5 to less than 10 years group (30 of 58 patients [51.7%]; *P* = .14). (Figure 3E). These data support the hypothesis that proximity to a recent childbirth may be associated with progression of ER-positive breast cancer to the liver, as previously reported in rodents and humans.^34,35^

## Discussion

The findings of this cohort study among individuals diagnosed with breast cancer at age 15 to 45 years in Utah during 1996 to 2017 suggest a new prognostic factor in addition to tumor stage and ER status in YOBC. Especially in stage I and II disease, we found the primary risk factor of progression to metastasis was not ER status, but rather a breast cancer diagnosis within 5 years of childbirth. These data also show that a recent childbirth was associated with poor prognosis in ER-positive disease, in which prognosis was associated with lymph node involvement, and in ER-negative disease, in which the poor prognosis of a postpartum diagnosis was independent of tumor stage. In fact, over time, patients with ER-positive PPBC had significantly worse distant metastasis–free survival than nulliparous patients diagnosed with ER-negative disease. These data suggest that, in YOBC, a postpartum diagnosis is the dominant risk factor of outcomes with more prognostic value than classic “good” prognostic tumor features, eg, stage I or II and ER-positive status. As such, time since recent childbirth needs to be factored into clinical care as an important prognostic factor.

Our finding that the PPBC within less than 5 years group had worse outcomes is consistent with several other epidemiological studies. A large meta-analysis^23^ of the association of time since recent childbirth with breast cancer among younger patients reported that the highest risk for mortality occurred within 5 years of childbirth, with patients with PPBC diagnosed within 1 year of childbirth having the highest risk of mortality (HR, 1.59; 95% CI 1.30-1.82) compared with nulliparous patients. The same meta-analysis^23^ demonstrated the long-lived duration of this postpartum risk, as mortality was statistically elevated for up to 6 years after recent childbirth (HR, 1.14; 95% CI, 0.99-1.25).

Overall, ER-negative breast cancers are increased in younger patients,^36,37,38,39^ which contributes to the poorer prognosis of younger patients in general. However, evidence that ER-negative tumors were enriched by parity in young patients or PPBC specifically has been weak,^36^ modest,^39,40^ or only observed with at least 3 pregnancies.^41^ In this UPDB study, we found a modest enrichment of ER-negative tumors in the PPBC within less than 5 years group, which is likely primarily due to the younger age of this group at time of diagnosis, rather than to an interaction between tumor subtype and recent childbirth. These observations may yield new insight into how postpartum biology increases incidence of metastasis. We argue that the timeline of disease occurrence (rapidly, within 5 years postpartum) and the lack of evidence for a shift in ER-positive vs ER-negative tumors in the PPBC group are consistent with postpartum biology promoting preexisting disease rather than initiating new disease. Furthermore, the increase in stage migration (ie, increased lymph node involvement) observed in the PPBC within less than 5 years group is unlikely to be due to delayed diagnosis, most patients were too young for routine screening. Instead, a model is emerging that is consistent with the tissue microenvironment of the postpartum breast being sufficient to promote early stage or quiescent disease to disseminated, metastatic disease.^26^

In rodents, the postpartum biology of weaning-induced mammary gland involution has been shown to promote breast cancer dissemination from the primary site through a microenvironmental, tumor cell extrinsic mechanism. Involution returns the lactation-competent gland to a quiescent, prepregnancy-like state via a wound-healing process^42,43^ that includes inflammation and active immune suppression,^44^ fibroblast activation,^45^ deposition of wound-like fibrillar collagens,^46,47^ and lymphangiogenesis.^30,48^ These stromal attributes are causally linked to metastasis in rodent models of PPBC.^30,45,49,50,51,52^ Potential relevance to humans is implicated by the recent demonstration that weaning-induced breast involution in healthy people is similar to rodent mammary gland involution with respect to rapidity of lobule loss, epithelial cell death, inflammation, collagen deposition, and lymphangiogenesis.^31,46^ The studies of normal breast involution not only define a postpartum biology consistent with tumor progression, but also provide evidence that tumors that evolve within the involuting gland are durably altered to a more invasive phenotype by the involution microenvironment.^30^ Comparative RNA sequence expression analyses on treatment-naive breast cancer tissues from young patients revealed PPBC tumors had enrichment of gene signatures associated with the normal process of weaning-induced breast involution compared with breast cancers diagnosed in young nulliparous patients.^46,53^ These and other studies reporting poor prognosis in breast cancers expressing gene signatures associated with classic weaning-induced mammary gland involution^53,54,55,56,57^ support a causative role for involution in breast cancer progression. With respect to further refining the postpartum window of risk for PPBC, one premise would be to delineate risk by time since weaning. A caveat to this approach is inferred by histomorphologic data obtained from the 2020 breast involution study by Jindal et al^31^ assessing healthy participants. In this study, half of the participants had breast lobule compositions at time of wean consistent with involution concurrent with lactation,^31^ a histology consistent with gradual weaning. Thus, the window of risk for PPBC could begin earlier than last date of nursing. Further research is needed to investigate potential causal links between postpartum lobule regression and breast cancer progression.

We also performed a first site of metastasis analysis and found that ER-positive disease was enriched in bone metastases and ER-negative disease was enriched in nervous system and brain metastases, consistent with previous reports.^58^ Previous studies in YOBC have identified bone as the most common site of metastasis in both ER-positive and ER-negative disease, with approximately 2-fold higher rates of metastasis to bone compared with liver.^59^ In this Utah cohort, we found liver metastasis rates to be essentially as high as bone, at 38% and 41%, respectively. We also observed only 4% lung metastasis, significantly lower levels than expected based on previous reports of 22% to 32% for all patients with breast cancer,^60^ and 25% in patients younger than 40 years.^59^ It is noteworthy that Utah has the lowest smoking rate in the country,^61,62^ suggesting smoking as a risk factor for breast cancer metastasis to the lung, as previously reported.^63,64^

We further analyzed site of metastasis by proximity to a recent childbirth; however, this analysis was restricted to patients with ER-positive disease due to sample size constraints. We found that patients diagnosed with ER-positive breast cancer within 5 years of childbirth had an increased incidence of liver metastasis compared with nulliparous patients, but not lung, brain or bone metastases. An earlier study of 564 patients with breast cancer diagnosed at age 45 years or younger showed an approximately 3.6-fold increase in liver metastasis in patients with PPBC diagnosed within less than 5 years after giving birth, with a continuous trend of up to 10 years.^34^ Based on the newly discovered biology of weaning-induced liver involution in rodents,^34^ which shares numerous tissue remodeling attributes with mammary gland involution, we propose that tissue-specific changes in the postpartum liver foster liver metastasis in patients with breast cancer with recent childbirth. In support, evidence that the human liver increases in size and anabolic metabolism output with pregnancy, followed by loss in size and increases in catabolic metabolism after weaning, has been recently reported^35^.

The high rate of liver metastasis in patients with PPBC likely reduces overall survival, as literature has shown that among patients with metastatic breast cancer, those with liver metastasis had shorter survival times. A 2003 study^65^ showed patients with breast cancer with liver metastasis had a median survival of 4.2 months, whereas a 2013 study^66^ reported that patients with breast cancer and bone-only metastasis had a median survival of 55.2 months. In a Danish nationwide registry-based cohort study,^67^ liver metastasis was associated with 43% risk of death within a year, whereas bone or lung metastases portended longer survival, with approximately 32% risk of death in the first year. Assessments of 5-year survival rates also indicate liver metastases to be especially deadly, with a 9% 5-year survival for liver metastasis,^68,69^ compared with 18% for lung^70^ and 10% for brain.^71^ Cumulatively, these studies highlight the need for improved treatment options for younger patients with metastatic breast cancer, especially liver metastasis. Future investigations of weaning-induced liver involution may provide insight into the liver metastatic niche and its therapeutic vulnerabilities.

Our study has many strengths. This is a statewide population-based study with a large sample size and long-term follow-up. The data source includes an in-depth cancer case-ascertainment system of the Utah Cancer Registry that ranks among the top in data quality and completeness. UPDB is one of the world’s largest sources of linked population-based information, with descendants’ information from the late 18th century. Therefore, we could readily link birth information and detailed cancer information, including first site of metastasis, to identify interactions between time since last childbirth and cancer end points.

### Limitations

This study has some limitations. We could not rigorously evaluate ERBB2 status by reproductive category due to the lack of ERBB2 clinical data for most patients in the Utah Cancer Registry. Also, the sample size of patients with stage III disease is relatively small, and our ability to assess associations is likely underpowered. Furthermore, small sample size also inhibited our ability to investigate whether first site of metastasis was associated with proximity to childbirth in patients with ER-negative disease. Another limitation is that our data lack numbers of pregnancies and live childbirths, breastfeeding metrics, time of wean, and lifestyle factors, such as body mass index, diet, physical activity, smoking, and alcohol, as well as detailed treatment information. However, we controlled for age differences in the comparisons and future research could address residual confounding factors through full propensity matching. Additionally, future research could take number of childbirths into consideration, since patients with PPBC more than 10 years after childbirth tend to have more children than those with PPBC within less than 5 years. Another limitation is that this study is based on a mostly non-Hispanic White population, as Utah has limited racial and ethnic diversity. Therefore, our results are more generalizable to the non-Hispanic White population.

## Conclusions

This cohort study found that a postpartum diagnosis was an independent risk factor for breast cancer progression and implicated birth-interval–associated biology, namely weaning-induced breast and liver involution, in the increased metastasis observed. Further research is needed to better understand the mechanisms by which a recent childbirth may promote poor outcomes in breast cancer. Nonetheless, irrespective of ER status, clinical consideration of time between recent childbirth and breast cancer diagnosis will increase accuracy of prognosis in patients with YOBC.

## Notes

Supplement. **eFigure 1.** Consort Diagram of the Analytic Cohort **eFigure 2.** Tumor Size by Parity Group **eTable 1.** UPDB’s Breast Cancer Cohort **eTable 2.** Frequency Distribution of Known First Site of Metastasis by Estrogen Receptor Status **eTable 3.** Multivariate Cox Proportional Regression Model for Time Since Recent Childbirth in Association with Distant Metastasis and Breast Cancer–Specific Mortality (Entire Cohort: Stage I, II, III, or Unknown)
