# Supplementary material for: Young-Onset Breast Cancer Outcomes by Time Since Recent Childbirth in Utah
Source: JAMA Netw Open. 2022 Oct 14;5(10):e2236763. doi: 10.1001/jamanetworkopen.2022.36763 (PMC9568799; doi:10.1001/jamanetworkopen.2022.36763)
Supplement: Supplement. — eFigure 1. Consort Diagram of the Analytic Cohort eFigure 2. Tumor Size by Parity Group eTable 1. UPDB’s Breast Cancer Cohort eTable 2. Frequency Distribution of Known First Site of Metastasis by Estrogen Receptor Status eTable 3. Multivariate Cox Proportional Regression Model for Time Since Recent Childbirth in Association with Distant Metastasis and Breast Cancer–Specific Mortality (Entire Cohort: Stage I, II, III, or Unknown) [file jamanetwopen-e2236763-s001.pdf]

Supplemental Online Content

Zhang Z, Bassale S, Jindal S, et al. Young-onset breast cancer outcomes by time since recent childbirth in Utah. *JAMA Netw Open*. 2022;5(10):e2236763. doi:10.1001/jamanetworkopen.2022.36763

eFigure 1. CONSORT Diagram of the Analytic Cohort
eFigure 2. Tumor Size by Parity Group
eTable 1. UPDB's Breast Cancer Cohort
eTable 2. Frequency Distribution of Known First Site of Metastasis by Estrogen Receptor Status
eTable 3. Multivariate Cox Proportional Regression Model for Time Since Recent Childbirth in Association with Distant Metastasis and Breast Cancer–Specific Mortality (Entire Cohort: Stage I, II, III, or Unknown)

This supplemental material has been provided by the authors to give readers additional information about their work.

© 2022 Zhang Z et al. *JAMA Network Open.*

eFigure 1. CONSORT Diagram of the Analytic Cohort

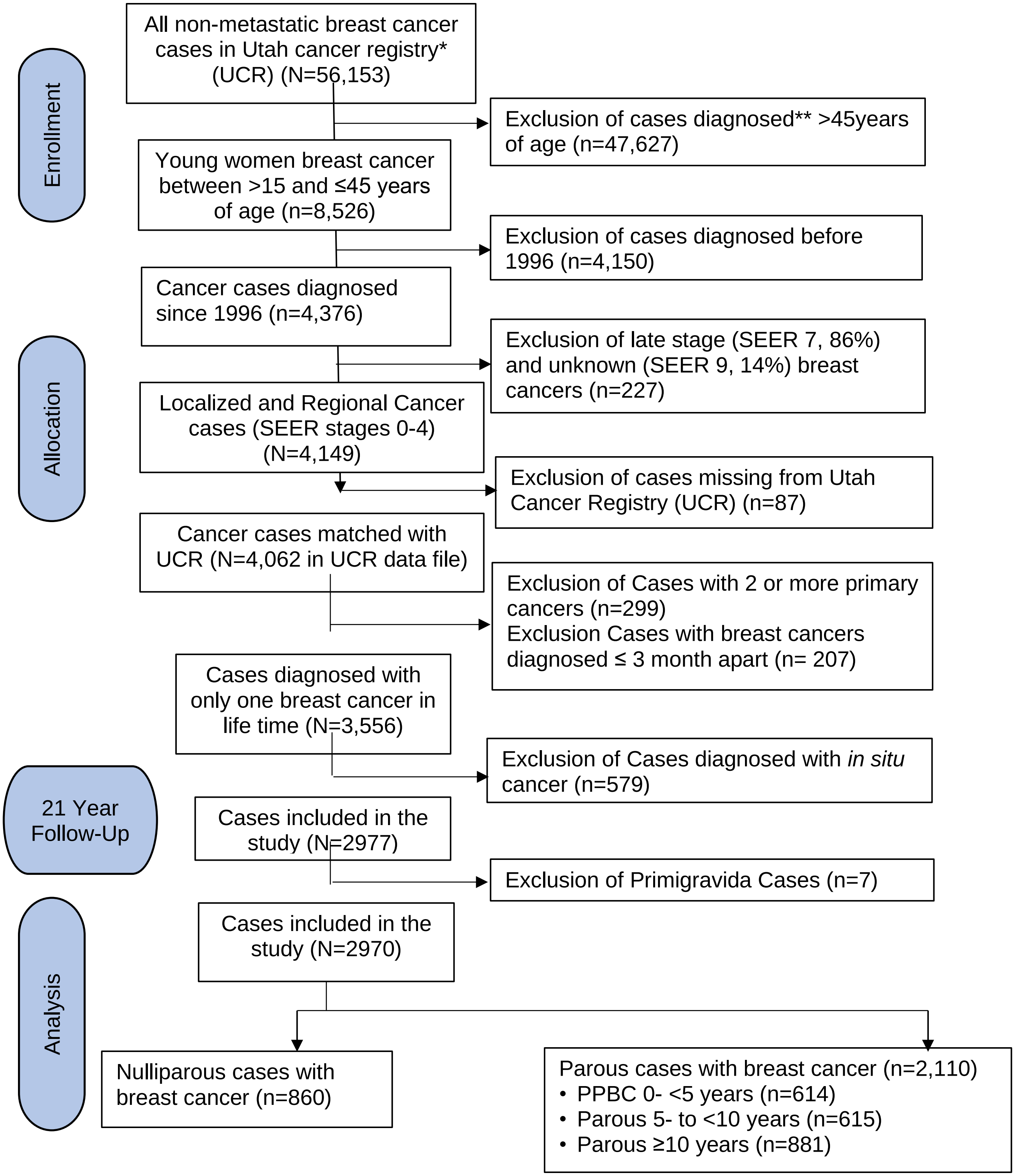

*Utah Cancer registry SEER DMS database – include SEER reportable cases, Utah resident at diagnosis, exclude cases only reported by VA hospital, exclude cases reported by other state registries, ambiguous diagnosis included

**2 additional cases < 15 years old excluded

© 2022 Zhang Z et al. *JAMA Network Open.*

This diagram depicts how cases from the Utah Population Database were selected for inclusion in our analytic cohort. In brief, non-metastatic, invasive breast cancer cases diagnosed between 01/01/1996 and 12/31/2017 were included in the cohort (N = 2970).

© 2022 Zhang Z et al. *JAMA Network Open.*

eFigure 2. Tumor Size by Parity Group

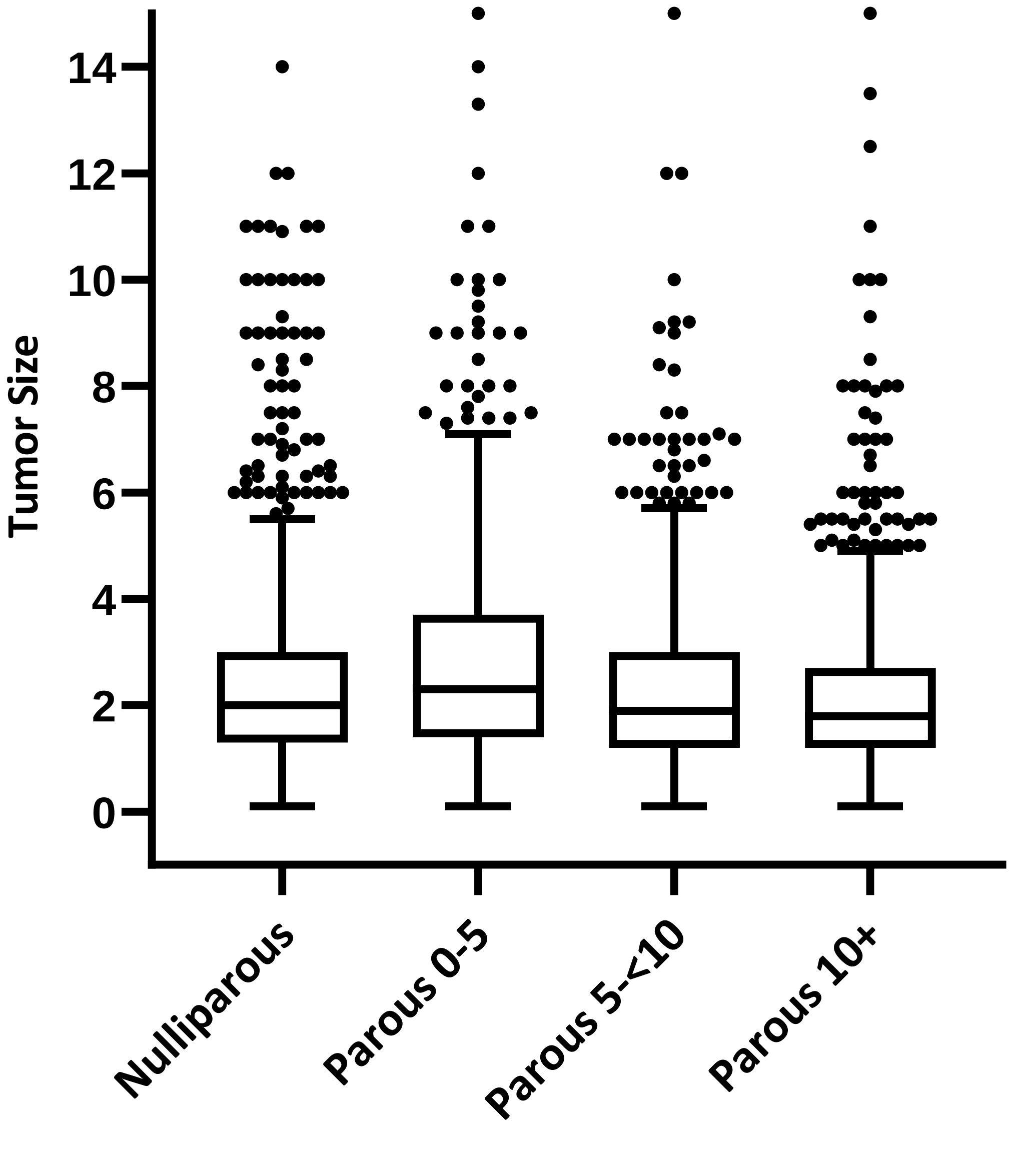

© 2022 Zhang Z et al. *JAMA Network Open.*

eTable 1. UPDB's Breast Cancer Cohort

	**Nulliparous (*N*=860, 29.1%)**		**PPBC <5 years (*N*=614, 20.6%)**		**PPBC 5-<10 years (*N*=615, 20.7%)**		**PPBC ≥10 (*N*=881, 29.6%)**	
	**No. (%)**		**No. (%)**		**No. (%)**		**No. (%)**	
**Mean age at diagnosis (SD)**	38.8 (5.7)		35.6 (5.0)		39.5 (4.1)		42.2 (2.7)	
**Median age in PPBC <5 years**	Age ≤ 36	Age > 36	Age ≤ 36	Age > 36	Age ≤36	Age > 36	Age ≤ 36	Age > 36
	(N=240)	(N=620)	(N=334)	(N=280)	(N=131)	(N=484)	(N=36)	(N=845)
**Estrogen status**								
ER+	169 (73.2)	473 (79.8)	223 (68.8)	214 (78.4)	94 (73.4)	373 (80)	27 (77.1)	635 (77.6)
ER-	62 (26.8)	120 (20.2)	101 (31.2)	59 (21.6)	34 (26.6)	93 (20)	8 (22.9)	183 (22.4)
Missing	14	29	10	7	3	18	1	27
**Lymph Node**								
Yes	108 (47)	275 (45.6)	206 (62.6)	154 (57.5)	59 (46.5)	198 (42)	16 (45.7)	335 (40.7)
No	122 (53)	328 (54.4)	123 (37.4)	114 (42.5)	68 (53.5)	273 (58)	19 (54.3)	488 (59.3)
No Nodes examined	15	19	5	12	4	13	1	22
**Tumor size**								
0.1—≤2.0 cm	112 (49.1)	331 (55.5)	126 (39.5)	133 (50.2)	72 (58.1)	248 (53)	19 (54.3)	475 (58.6)
>2.0—≤5.0 cm	88 (38.6)	217 (36.4)	145 (45.5)	94 (35.5)	37 (29.8)	183 (39.1)	14 (40)	292 (36)
>5.0 cm	28 (12.3)	48 (8.1)	48 (15)	38 (14.3)	15 (12.1)	37 (7.9)	2 (5.7)	43 (5.3)
Missing	12	24	15	15	7	16	1	35
**Stage**								
I	84 (40.2)	246 (49.4)	71 (26.7)	87 (36.9)	52 (47.7)	194 (46.4)	16 (57.1)	375 (53)
II	93 (44.5)	203 (40.8)	136 (51.1)	105 (44.5)	33 (30.3)	174 (41.6)	9 (32.1)	261 (36.9)
III	32 (15.3)	49 (9.8)	59 (22.2)	44 (18.6)	24 (22)	50 (12)	3 (10.7)	71 (10)
Missing	36	124	68	44	22	66	8	138
**Biologic subtype**								

© 2022 Zhang Z et al. *JAMA Network Open.*

Luminal A (ER+, PR+/-, Her2 neu-)	52 (51)	159 (71)	83 (53.5)	91 (65)	35 (61.4)	161 (72.2)	13 (76.5)	251 (68.2)
Luminal B (ER+, PR+/-, Her2 neu+)	27 (26.5)	34 (15.2)	37 (23.9)	25 (17.9)	10 (17.5)	26 (11.7)	2 (11.8)	50 (13.6)
Her2 neu positive (ER-, PR-)	10 (9.8)	9 (4)	13 (8.4)	8 (5.7)	6 (10.5)	14 (6.3)	0 (0)	18 (4.9)
Triple negative	13 (12.7)	22 (9.8)	22 (14.2)	16 (11.4)	6 (10.5)	22 (9.9)	2 (11.8)	49 (13.3)
Unknown Her2 neu	136	384	175	139	73	258	19	474
Unknown/Other	2	12	4	1	1	3	0	3
**Year of Diagnosis**								
1996-1998	23 (9.4)	71 (11.4)	31 (9.3)	18 (6.4)	15 (11.5)	45 (9.3)	4 (11.1)	96 (11.4)
1999-2004	46 (18.8)	162 (26.1)	61 (18.3)	58 (20.7)	28 (21.4)	92 (19)	11 (30.6)	210 (24.9)
2005-2017	176 (71.8)	389 (62.5)	242 (72.5)	204 (72.9)	88 (67.2)	347 (71.7)	21 (58.3)	539 (63.8)
**Patients with Metastasis**	33 (13.5)	67 (10.8)	78 (23.4)	40 (14.3)	15 (11.5)	65 (13.4)	7 (19.4)	93 (11)

Note:
Abbreviations: PPBC, Postpartum Breast Cancer; No., number of patients per group; ER, estrogen receptor; PR, progesterone receptor;

© 2022 Zhang Z et al. *JAMA Network Open.*

eTable 2. Frequency Distribution of Known First Site of Metastasis by Estrogen Receptor Status

	ER+ (n=2203)					ER- (n=658)				
**Known First Site of Metastasis**	**Total n of Metastasis**	**Nulliparous**	**PPBC 0-5**	**Parous 5-<10**	**Parous ≥10**	**Total n of Metastasis**	**Nulliparous**	**PPBC 0-5**	**Parous 5-<10**	**Parous ≥10**
Bone	130 (46%) [c]	36 (28%) [e]	31 (24%) [e]	30 (23%) [e]	33 (25%) [e]	22 (25%) [d]	3 (14%) [e]	7 (32%) [e]	5 (23%) [e]	7 (32%) [e]
Liver	108 (38%) [c]	28 (26%) [e]	38 (35%) [e]	18 (17%) [e]	24 (22%) [e]	33 (37.5%) [d]	8 (24%) [e]	12 (36%) [e]	4 (12%) [e]	9 (28%) [e]
Brain + Nervous System	32 (11%) [c]	11 (34%) [e]	12 (38%) [e]	7(22%) [e]	2 (6%) [e]	29 (33%) [d]	3 (10%) [e]	10 (34%) [e]	6 (21%) [e]	10 (35%) [e]
Lung	12 (4%) [c]	2 (17%) [e]	2 (17%) [e]	3 (25%) [e]	5 (42%) [e]	3 (3%) [d]	1 (33%) [e]	0	0	2 (67%) [e]
Kidney	0	0	0	0	0	1 (1%) [d]	0 (14%) [e]	0	0	1 (100%) [e]
Total	282 (12.8%) [a]	77 (27%) [e]	83 (29%) [e]	58 (21%) [e]	64 (23%) [e]	88 (13.4%) [b]	15 (17%) [e]	29 (33%) [e]	15 (17%) [e]	29 (33%) [e]

a. Percentages based on total number of participants with ER+ (n=2203)
b. Percentages based on total number of participants with ER- (n=658)
c. Percentages based on total number of participants with known first site of metastasis in ER+ group (n=282)
d. Percentages based on total number of participants with known first site of metastasis in ER- group (n=88)
e. Percentages on number of participants with known first site of metastasis by estrogen receptor status and site of metastasis (row percentage).

© 2022 Zhang Z et al. *JAMA Network Open.*

eTable 3. Multivariate Cox Proportional Regression Model for Time Since Recent Childbirth in Association with Distant Metastasis and Breast Cancer–Specific Mortality (Entire Cohort: Stage I, II, III, or Unknown)

		Distant–Metastasis				Breast Cancer-Specific Mortality			
**Variable**	**Levels**	**Hazard Ratio**	**95% HR CI**	**P value**	**Overall P**	**Hazard Ratio**	**95% HR CI**	**P value**	**Overall P**
Time since recent childbirth	Nulliparous	Reference			0.02	Reference			0.039
	PPBC <5	1.5	1.2-2.0	0.002		1.5	1.1-2.1	0.004	
	Parous 5 to <10	1.2	0.9-1.6	0.25		1.3	0.9-1.7	0.110	
	Parous >=10	1.1	0.8-1.5	0.57		1.2	0.9-1.6	0.315	
Diagnosis Year	1996-1998	Reference			<.001	Reference			<.001
	1999-2004	0.8	0.6-1.1	0.15		0.8	0.6-1.1	0.109	
	2005-2017	0.5	0.4-0.7	<.001		0.4	0.3-0.6	<.001	
Diagnosis Age		1	0.96-1.0	0.06	0.06	1.0	0.97-1.0	0.644	0.64
AJCC Stage	I	Reference			<.001	Reference			<.001
	II	2.6	1.9-3.6	<.001		2.6	1.9-3.7	<.001	
	III	5.9	4.1-8.5	<.001		7.3	4.9-10.7	<.001	
	Unknown	4.5	3.3-6.2	<.001		5.1	3.7-7.1	<.001	
ER	Positive/Elevated	Reference			0.88	Reference			0.001
	Negative/Normal	1	0.8-1.2	0.8416		1.6	1.2-2.0	<.001	
	Unknown	0.9	0.5-1.5	0.6236		0.96	0.6-1.6	0.888	

© 2022 Zhang Z et al. *JAMA Network Open.*
